# Molting incidents of *Hyalomma* spp. carrying human pathogens in Germany under different weather conditions

**DOI:** 10.1186/s13071-024-06175-y

**Published:** 2024-02-19

**Authors:** Lidia Chitimia-Dobler, Andrea Springer, Daniel Lang, Alexander Lindau, Katrin Fachet, Gerhard Dobler, Ard M. Nijhof, Christina Strube, Ute Mackenstedt

**Affiliations:** 1 Bundeswehr Institute of Microbiology, Neuherbergstrasse 11, 80937 Munich, Germany; 2 Fraunhofer Institute of Immunology, Infection and Pandemic Research, Penzberg, Germany; 3 Institute for Parasitology, Centre for Infection Medicine, University of Veterinary Medicine Hannover, Buenteweg 17, 30559 Hanover, Germany; 4 Department of Parasitology, Institute of Biology, University of Hohenheim, Emil-Wolff-Strasse 34, 70599 Stuttgart, Germany; 5 Institute for Parasitology and Tropical Veterinary Medicine, Freie Universität Berlin, Robert-Von-Ostertag-Str. 7, 14163 Berlin, Germany; 6 Veterinary Centre for Resistance Research, Freie Universität Berlin, Robert-Von-Ostertag-Str. 8, 14163 Berlin, Germany

**Keywords:** *Hyalomma marginatum*, *Hyalomma rufipes*, Weather conditions, Tick-borne pathogens, Germany

## Abstract

**Background:**

*Hyalomma marginatum* and *H. rufipes* are two-host tick species, which are mainly distributed in southern Europe, Africa to central Asia but may also be found in Central and Northern Europe through introduction by migratory birds.

**Methods:**

Ticks were collected while feeding or crawling on animals and humans, or from the environment, in different regions in Germany, between 2019 and 2021 in a citizen science study and from 2022 to 2023 in the wake of this study.

**Results:**

From 2019 to 2023, a total of 212 *Hyalomma* adult ticks were detected in Germany. This included 132 *H. marginatum* and 43 *H. rufipes* ticks sent to research institutions and 37 photographic records that were only identified to genus level. The number of detected ticks varied over the years, with the highest number of 119 specimens recorded in 2019, followed by 57 in 2020. Most of the specimens were collected from horses, while some were collected from other animals, humans or found crawling on human clothes or other objects inside or outside houses. The screening of 175 specimens for Crimean-Congo hemorrhagic fever virus and of 132 specimens for *Babesia*/*Theileria* spp. by PCR gave negative results, while human-pathogenic *Rickettsia* were detected in 44% (77/175) of the total samples. Subsequent amplicon sequencing and phylogenetic analysis of representative samples determined the species of 41 *Rickettsia aeschlimannii* and one *R. slovaca* sequences.

**Conclusions:**

Analysis of climatic factors indicated a significantly higher probability of *Hyalomma* occurrence at locations with higher average spring temperature during the years 2019 and 2020 compared to randomly generated pseudo-absence locations. Dry and hot conditions probably facilitated *Hyalomma* nymphs’ survival and molting into adults during these years.

**Graphical Abstract:**

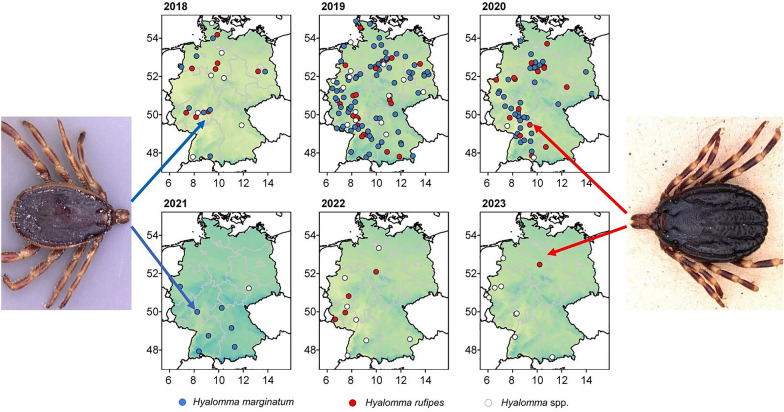

**Supplementary Information:**

The online version contains supplementary material available at 10.1186/s13071-024-06175-y.

## Background

The family Ixodidae is divided into two groups, the Prostriata and the Metastriata, which currently comprise 762 species assigned to 15 extant genera and two extinct genera [1]. In Europe, ixodid tick species belong to five genera: *Ixodes* (Prostriata) as well as *Dermacentor*, *Haemaphysalis*, *Hyalomma* and *Rhipicephalus* (Metastriata) [2]. Ticks are hematophagous parasites and can be specialists, adapted to one host or a narrow group of hosts, e.g. *Ixodes lividus* (Koch, 1844) to the sand martin (*Riparia riparia*), or generalists with a wide host range such as *Ixodes ricinus* (Linnaeus, 1758). Generally, tick larvae and nymphs usually infest small mammals and birds, while adults prefer large mammals [3]. Larvae and nymphs of tick species belonging to the genera *Ixodes*, *Haemaphysalis* and *Hyalomma* can parasitize ground-feeding and ground-breeding birds [4–6], while the genera *Dermacentor* and *Rhipicephalus* usually do not parasitize birds [7, 8].

Generally, ticks are able to move over short distances only. *Ixodes scapularis* nymphs and adults move only 2–3 m and 5 m, respectively [9], while *Dermacentor reticulatus* adults were reported to cover an average distance of approximately 60 cm in 7 weeks [10]. Ticks can be dispersed over larger distances by movements of their hosts, such as livestock or wild animals and migratory birds [11]. Migratory birds transport ticks during their annual migrations across international borders and continents [12]. The European bird population includes several billion birds that migrate annually during spring to their breeding grounds in Europe and return in autumn to their non-breeding grounds in southern regions [13].

Birds can carry immatures of at least two species of the genus *Hyalomma*, *Hyalomma* (*H.*) *marginatum* and *Hyalomma rufipes*. Both are two-host ticks of which the immatures feed for up to 4 weeks on hosts, thus allowing transportation and further spread into new geographical areas [14]. The role of migratory birds in the epizootology and epidemiology of ticks and tick-borne pathogens has received increased attention in recent years [15–18]. Prominent examples are the recent introduction of *H. marginatus* and *H. rufipes* into Germany [16, 19, 20] and Sweden [17]. Migratory birds can disseminate their associated ticks and related pathogens over long distances and in areas where medium or large mammals’ access is limited [21, 22], and across natural geographic barriers, such as oceans or deserts [23].

Hoogstraal’s work [24] has shown for the first time that the migratory routes between Africa and Europe serve as routes of dissemination of ticks belonging to the genus *Hyalomma*. The increased reporting of *Hyalomma* ticks during the last years in Central and North Europe raised speculations that these usually (sub-)tropical ticks may establish populations in areas outside of their current distribution because of climate changes [25].

However, Buczek [26] observed that a high relative humidity of 90% hampers the embryonic development of *H. marginatum* maintained at 25 ℃, with high rates of embryo mortality, abnormally hatched larvae and development of larvae with morphological anomalies. Regarding *H. rufipes*, Theiler [27] observed that temperature alone does not appear to be a restrictive factor for the distribution of this tick species, which commonly occurs in semiarid regions of Africa and in savannahs with a long, hot, severe dry season, but is less common or absent in areas with > 760 mm of annual rainfall, semitropical zones, humid seacoasts and other zones with high relative humidity, despite low annual rainfall.

*Hyalomma* larvae and nymphs are regularly found on migratory birds and therefore serve as excellent examples of tropical or sub-tropical tick species molting from the nymphal to the adult stage under suitable weather conditions outside their usual distribution area [15–18]. The last years (2018–2020) exhibited unusually high temperature conditions in parts of Europe. Therefore, there is increased concern that invading tick species, and particularly *Hyalomma* ticks, might also introduce pathogens such as Crimean-Congo hemorrhagic fever virus (CCHFV), Alkhumra virus, *Rickettsia aeschlimannii* or *Babesia/Theileria* spp.

A previous study, showed that *Hyalomma* ticks were detected in increasing numbers in Germany in 2018, a year with high temperatures and low rainfall [16]. To pursue this observation further, we conducted a citizen science study from 2019 to 2021 to relate the occurrence and reporting of *Hyalomma* ticks in Germany with weather conditions. The aim was to identify weather conditions that bear a higher risk of development of engorged nymphs into adults and of pathogen transmission.

## Methods

### Citizen-science call

After a high number of *Hyalomma* spp. was reported in 2018 in Germany, the Department of Parasitology at the University of Hohenheim in Stuttgart, southern Germany, released a call to send in *Hyalomma* ticks as well as ticks of unusual appearance at the end of February 2019 which ended by the end of 2021. The respective press releases were circulated in various regional and national media; additionally, a website was designed with further information. All media releases included pictures to help citizens distinguish between different tick genera. Along with the ticks, citizens were asked to provide information on the date and location of collection [Global Positioning System (GPS) data or postal code], the involvement of potential hosts and details about the circumstances under which the tick was discovered. To activate participates’ motivation, citizens received feedback and were informed about the identity of the tick species that they submitted.

### Tick collection and identification

Ticks were collected feeding or crawling on animals and humans or from the environment in different regions and districts in Germany from 2019 to 2023 (Table 1). Individual ticks were shipped directly to the University of Hohenheim, the University of Veterinary Medicine Hannover, the Bundeswehr Institute of Microbiology, the Institute for Parasitology and Tropical Veterinary Medicine of the Freie Universität Berlin or to Public Health offices. Ticks were identified based on morphological characters according to Apanaskevich and Horak [28] and were further screened for the presence of pathogens. Furthermore, photographic records were identified to genus level and included in statistical analysis.

**Table 1:** Characteristics of Hyalomma species included in the study, 2019–2023

Year	Federal state	Tick species		Shipment institution
		*Hyalomma marginatum*	*Hyalomma rufipes*	
2019	Baden-Wuerttemberg	7 (1 m, 6 f)	2 (1 m, 1 f)	Department of Parasitology, University of Hohenheim, Stuttgart
	Bavaria	9 (5 m, 4 f)	1 (f)	
	Brandenburg	8 (3 m, 5 f)	1 (1 m)	
	Hamburg	1 (m)	–	
	Hesse	1 (m)	2 (1 m, 1f)	
	Lower Saxony	6 (4 m, 2 f)	3 (2 m, 1f)	
	North Rhine-Westphalia	11 (7 m, 4 f)	2 (m)	
	Rhine-Palatine	9 (5 m, 4 f)	2 (m)	
	Saarland	2 (m)	–	
	Saxony	1 (f)	–	
	Saxony- Anhalt	2 (f)	–	
	Schleswig–Holstein	4 (2 m, 2 f)	1 (m)	
	Thuringia	–	1 (m)	
	North Rhine-Westphalia	–	1 (f)	Institute for Parasitology and Tropical Veterinary Medicine of the Freie Universität Berlin
	Baden-Wuerttemberg	1 (m)	–	University of Veterinary Medicine Hannover
	Brandenburg	1 (f)	–	
	Lower Saxony	6 (4 m, 2 f)	2 (1 m, 1 f)	
	North Rhine-Westphalia	2 (1 m, 1 f)	–	
	Rhine-Palatine	3 (1 m, 2 f)	1 (m)	
	Saarland	1 (f)		
	Thuringia		2 (m)	
	Brandenburg	3 (m)	–	Bundeswehr Institute of Microbiology
	Bavaria		1 (m)	
	Hesse	1 (f)		
	North Rhine-Westphalia	4 (m)		
2020	Baden-Wuerttemberg	9 (5 m, 4 f)	4 (m)	Department of Parasitology, University of Hohenheim, Stuttgart
	Bavaria	1 (f)	1 (f)	
	Brandenburg	1 (m)	–	
	Hesse	6 (4 m, 2 f)	–	
	Lower Saxony	8 (6 m, 2 f)		
	North Rhine-Westphalia	6 (4 m, 2 f)		
	Rhine-Palatine	3 (2 m, 1 f)	1 (f)	
	Saxony	1 (m)	1 (m)	
	Schleswig–Holstein		1 (m)	
	Thuringia	1 (m)		
	Hesse	1 (m)	1 (m)	University of Veterinary Medicine Hannover
	Lower Saxony	7 (1 m, 6 f)	2 (1 m, 1 f)	
	North Rhine-Westphalia	–	1 (f)	
2021	Baden-Wuerttemberg	2 (m)	–	Department of Parasitology, University of Hohenheim, Stuttgart
	Bavaria	6 (5 m, 1 f)	–	
	North Rhine-Westphalia	1(m)	–	
	Rhine-Palatine	1 (f)	–	
2022	North Rhine-Westphalia		1 (m)	
	Lower Saxony	–	2 (m)	Department of Parasitology, University of Hohenheim, Stuttgart
	Rhine-Palatine	–	2 (1 m, 1 f)	University of Veterinary Medicine Hannover
2023	Lower Saxony	–	1 (female)	University of Veterinary Medicine Hannover

*m* male, *f* female, *A* adult

### Nucleic acid extraction and PCR

Total nucleic acid was extracted using the MagNA Pure LC RNA/DNA Kit (Roche, Mannheim, Germany) in a MagNA Pure LC instrument according to the manufacturer’s instructions. The extracted total nucleic acid was stored at – 80 ℃ until use.

Ticks were tested for CCHF virus using a previously published real-time RT-PCR [29] and *Rickettsia* spp. DNA using a pan-*Rickettsia* real-time PCR [30], followed by a 23S-5S intergenic spacer region PCR [31] for all positive samples with a Ct value < 35 to identify the *Rickettsia* species. Furthermore, the ticks were tested for the presence of *Babesia* spp. and *Theileria* spp. using a conventional PCR amplifying part of the 18S rRNA gene with primers BJ1 and BN2 [32], as previously described by Springer et al. [33].

### Sequence analysis of rickettsial 23S-5S intergenic spacer region

Rickettsial 23S-5S intergenic spacer region amplicon sequences were converted to FASTQ format using Tracy [34] basecall, both reads were searched against the GenBank Nucleotide database *Rickettsia* partition using the BLASTN algorithm (-evalue 1e-100 -dust no -soft_masking no -max_target_seqs 100), and subsequent hits were filtered using python binning (scikit-learn KBinsDiscretizer), ranking the results by bitscore to define the optimal reference sequence (best bitscore) and closest homologs (top bitscore bin) to be collected for subsequent phylogenetic analysis. Subsequently, the determined closest reference for each isolate was used to infer consensus sequences by variant calling using bwa-mem [35], freebayes [36] and bcftools [37] masking positions with coverage < 1. Sequences with insufficient coverage were discarded. The resulting 41 sequences were compared to 59 rickettsial sequences (non-redundant, top bitscore bin set determined earlier) using multiple sequence alignment with the MAFFT G-INSI [38] algorithm. Phylogenetic inference using Maximum Likelihood was performed testing branches by SH-like aLRT and aBayes parametric test of the topology determined with the best fitting substitution model HKY + F + I (based on corrected and regular Akaike information criterion as well as Bayesian information criterion) using ModelFinder implemented in IQ-TREE 1.6.12. Support for the topologies was tested by bootstrapping over 1000 replicates. Species classification of the 42 isolates (accession numbers: OZ002747-OZ002787) was based on the taxonomic affiliation of existing GenBank entries in the respective phylogenetic tree clades of each isolate presented in the Additional file 2: Figure S1.

### Investigation of climate factors associated with *Hyalomma* occurrence

The relationship of climate with the probability of *Hyalomma* occurrence was investigated using R v. 4.2.1 [39]. Gridded climate data, including mean temperature, precipitation sum and de Martonne drought index [40], for the spring (March–May) and summer (June–August) periods of each study year were retrieved from the Climate Data Center of the German Weather Service [41–43] using the package *rdwd* v. 1.8.0 [44]. The data were available in a 1 × 1 km resolution but were aggregated to a 5 × 5 km grid for the analysis to account for the relative inaccuracy of *Hyalomma* occurrence records as often only the postal code for a record was available. Elevation data were obtained at a 2.5 min resolution from the Worldclim database [45] using the package *raster* v. 3.6–26 [46]. The environmental variables were associated with the *Hyalomma* presence records and 1000 “pseudo-absence” locations randomly generated using the R package *biomod2* v. 4.2–4 [47]. Furthermore, generalized linear models (GLMs) with binomial error structure were constructed to assess the relationship between the climate variables and the probability of *Hyalomma* occurrence vs. pseudo-absence. As the variables precipitation and aridity index were strongly correlated, only the aridity index of the spring and summer period was included in the models in addition to elevation and mean spring and summer temperature. The analysis, including pseudo-absence sampling and GLM construction, was conducted separately for the years 2019 and 2020 as well as for previously published records from 2018 [16] and was repeated ten times each. Due to the low number of received ticks from 2021 onwards, a meaningful analysis was not possible for these years.

## Results

### *Hyalomma* occurrence in Germany during 2019–2023

**Fig. 1:**
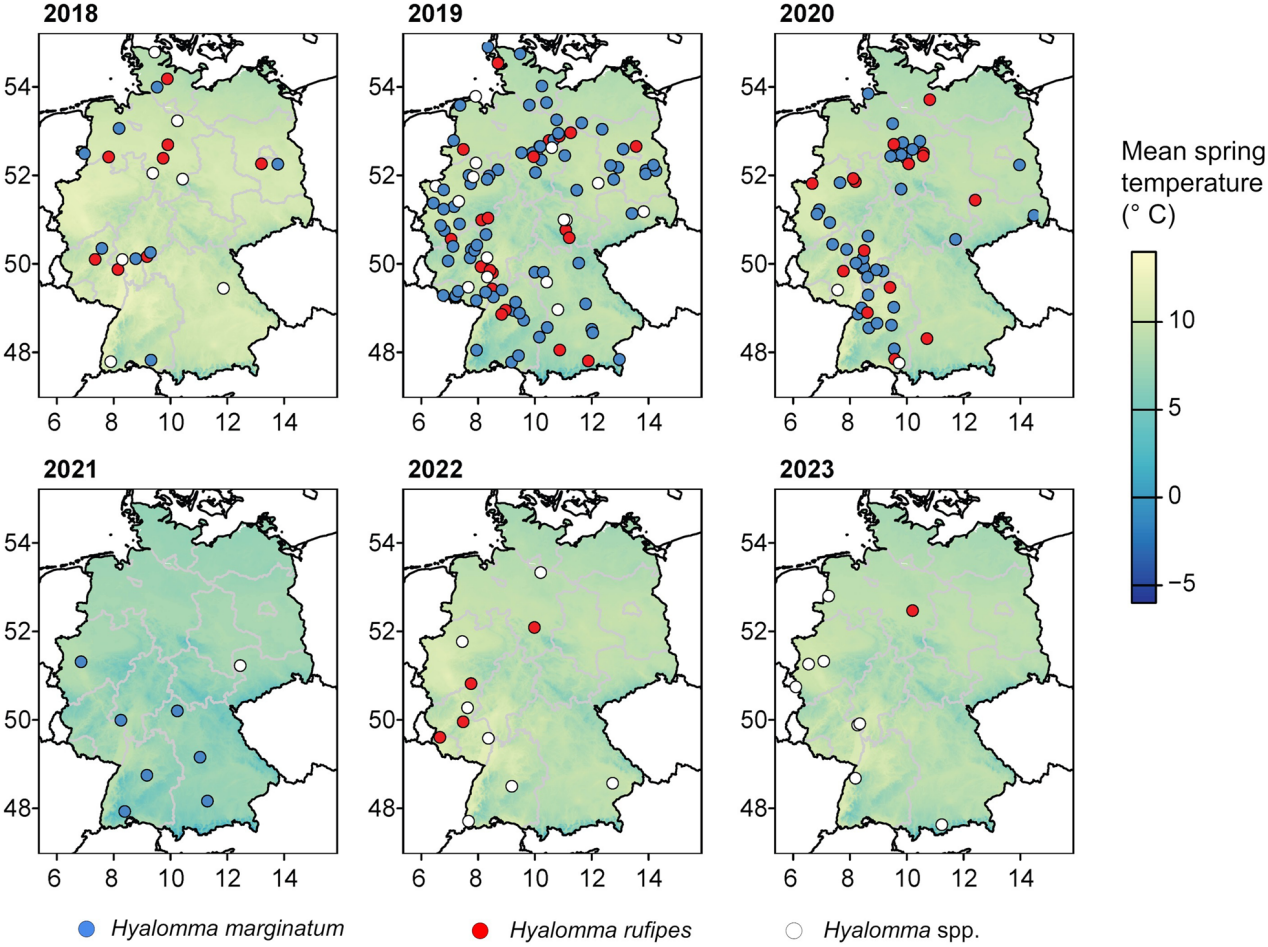
*Hyalomma* spp. findings in Germany in the frame of a Citizen Science study between 2018 and 2023. Locations in 2018 include records from Chitimia-Dobler et al. (2019). The color gradient of the maps indicates average spring temperature (Databasis: German Weather Service [Deutscher Wetterdienst], gridded data reproduced graphically)

In the current study, data on a 5-year surveillance of the introduction of two *Hyalomma* spp. by migratory birds into Germany are presented. In total, 212 adult *Hyalomma* specimens were identified, of which 175 specimens were shipped directly to one of the involved institutions and could be identified to species level and tested for the most relevant pathogens. Two *Hyalomma* species were identified: *H. marginatum* (132/175, 75.4%) and *H. rufipes* (43/175, 24.6%). *Hyalomma* ticks were found in 13 of the 16 German federal states (Table 1, Fig. 1).

In 2019, 119 adult specimens were reported (82 *H. marginatum*, 22 *H. rufipes*, 16 *Hyalomma* spp.) from 13 of the 16 German federal states, predominantly from North Rhine Westfalia, Lower Saxony and Rhineland Palatinate (Table 1). In 2020, the total number of reported adult ticks was lower than in 2019 (57 specimens, 40 *H. marginatum*, 15 *H. rufipes*, two *Hyalomma* spp.) from ten German federal states. In this year, most specimens were found in Lower Saxony, followed by Baden-Wuerttemberg, North Rhine-Westphalia and Hesse and only few specimens from other federal states (Table 1). In the wake of the citizen science study from 2022 to 2023, only a few adults of *Hyalomma* were reported, ten *H. marginatum* and one *Hyalomma* spp. in 2021, five *H. rufipes* and 11 *Hyalomma* spp. in 2022, one *H. rufipes* female and eight *Hyalomma* spp. in 2023 (Table 1).

**Fig. 2:**
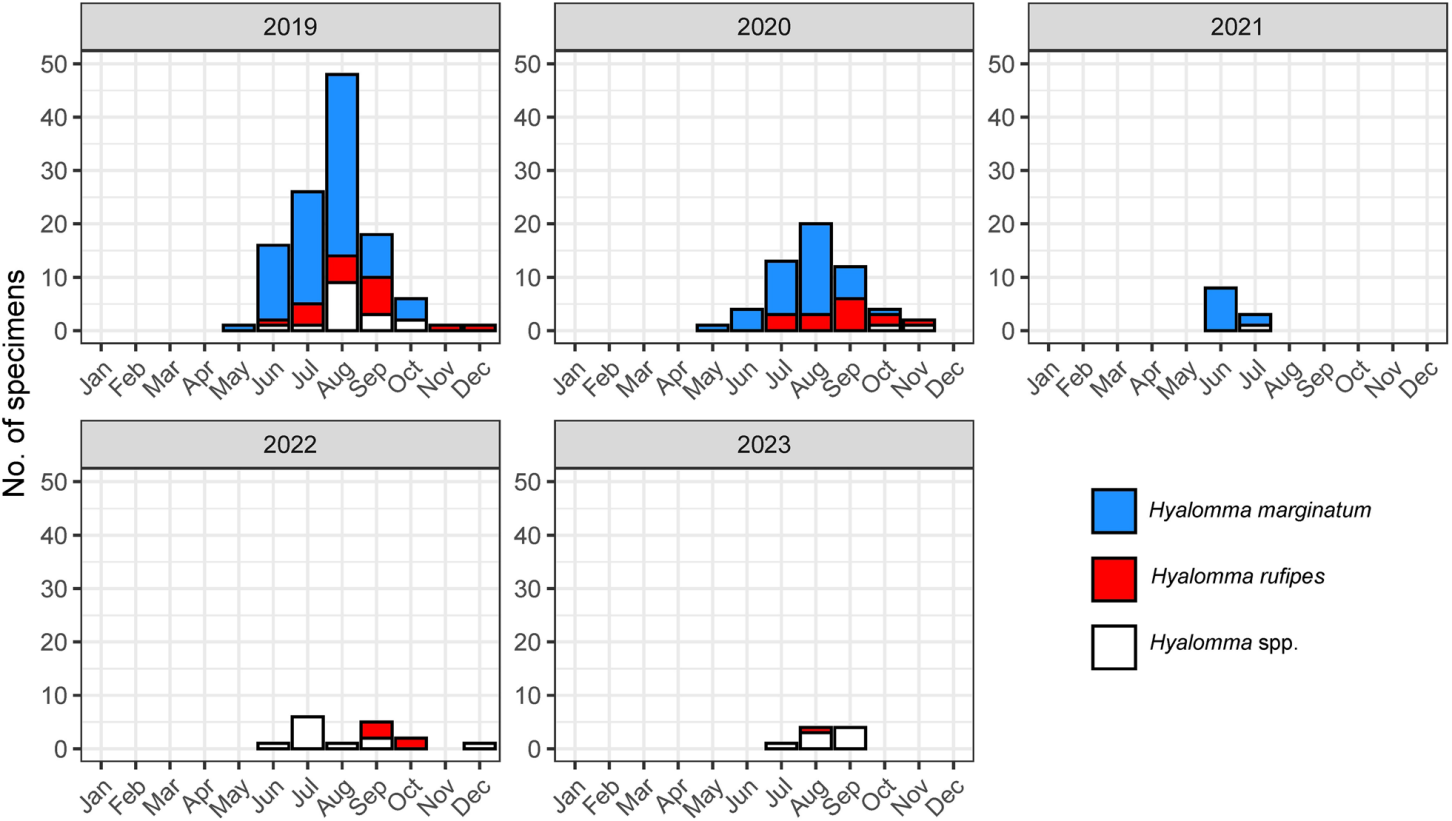
Monthly *Hyalomma* spp. occurrence in Germany as determined in the frame of a Citizen Science study between 2019 and 2023

All *Hyalomma* specimens were detected between the months of May and December. In the years 2019 and 2020, findings peaked in the month of August (Fig. 2).

*Hyalomma* specimens were mostly collected from horses (132) and other animals (two cows, one donkey, four dogs). Surprisingly, also 11 specimens were collected from humans. In most of the cases ticks were feeding on the respective host, but 69 specimens were found crawling on animals, human clothes or other belongings.

### Pathogen prevalence

All received specimens tested negative for CCHFV. *Rickettsia* spp. was detected in 77 (44.0%) of all tested *Hyalomma* ticks by PCR. Due to the low amount of DNA (Ct ≥ 35), 20/77 (25.9%) samples could not be amplified and sequenced. *Rickettsia aeschlimannii* was identified by sequencing of the 23S-5S intergenic spacer region from 51 *Hyalomma* specimens, 30/132 (22.7%) *H. marginatum* and 21/43 (48.8%) *H. rufipes* ticks. Only one sequence was identified as *Rickettsia slovaca*, in a *H. marginatum* collected from a horse in Baden-Württemberg. Of 132 samples available for *Babesia* and *Theileria* testing, none were positive.

### Relationship of *Hyalomma* occurrence with climate data

*Hyalomma* spp. occurrence was noted at 212 unique locations, 23 in 2018 (including records previously published by Chitimia-Dobler et al. [16]), 107 in 2019, 54 in 2020, 8 in 2021, 11 in 2022 and 9 in 2023 (Fig. 1).

**Fig. 3:**
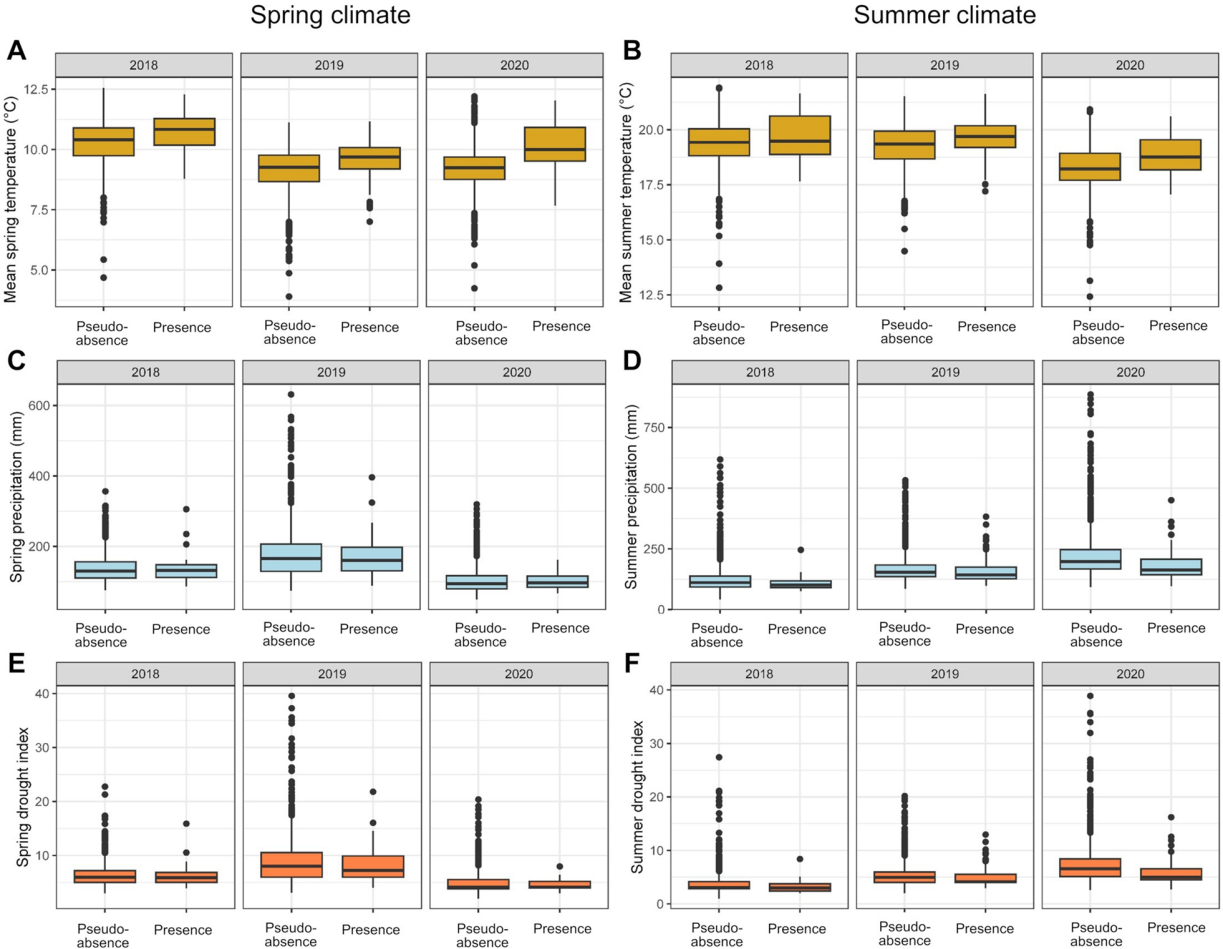
Spring (**A**–**E**) and summer (**B**–**F**) climate in Germany during 2018–2020 at locations where *Hyalomma* spp. were found vs. 1000 randomly generated pseudo-absence locations (Databasis: German Weather Service [Deutscher Wetterdienst]). Values from one of ten replicate analyses are shown

The difference in climate variables at *Hyalomma* presence locations vs. an exemplary set of 1000 pseudo-absence locations is shown in Fig. 3. During the years 2019 and 2020, when most *Hyalomma* specimens were received, a higher mean spring temperature was significantly associated with a higher probability of *Hyalomma* occurrence. This was consistent across all ten analysis replicates, with a mean model estimate of 1.4 (mean standard deviation [SD]: 0.35) for 2019 and of 0.85 (mean SD: 0.25) for 2020, i.e. 4.2 and 2.3 times higher odds of *Hyalomma* occurrence, respectively, per 1 ℃ increase in mean spring temperature. In fact, the mean spring temperature at locations where *Hyalomma* occurred in 2019 was 9.6 ℃ ,compared to the country-wide average of 9.1 ℃ during this year, and 10.1 ℃ in 2020 compared to a country-wide average of 9.2 ℃.

In contrast, no significant associations were found regarding spring or summer drought index. Elevation and mean summer temperature were identified as significant variables in only one respectively six of the ten replicate analyses for 2019 (Additional file 1: Table S1), whereby summer temperature showed a negative estimate, i.e. higher temperatures were associated with a lower probability of *Hyalomma* occurrence.

## Discussion

Ticks can be introduced into a territory by uncontrolled movements of livestock or the seasonal migratory behavior of birds [48]. Besides biotic factors (available hosts), environmental conditions, especially temperature and humidity ranges, are important abiotic factors for ticks to survive and establish populations in new geographic areas. Incidental reporting in new geographical areas of ticks, and especially of both *Hyalomma* species, is a well-known feature and has been documented by Hoogstraal et al. [4]. For several species in the Nearctic [49, 50] and Palearctic regions [51, 52], the incidental occurrence or establishment of populations has also been documented, mostly connecting this spread with warmer temperatures [53].

*Hyalomma marginatum* and *H. rufipes* are two-host ticks, and the immatures spend up to 28 days on a host, which facilitates long distance transport by migratory birds [4, 14]. During the last years, many countries in Central and Northern Europe reported the presence of an unusually high number of *H. marginatum* and to a lesser extent of *H. rufipes* [15, 17, 18]. The same phenomenon was observed in Germany in 2018 [16], 2019 and 2020, with decreasing numbers thereafter. The years 2018 to 2020 were characterized by unprecedented warm and dry conditions in Central Europe [54]. Nevertheless, due to the potential bias caused by media attention in the frame of the citizen science study, which ended in 2021, caution should be exerted when comparing the *Hyalomma* numbers between different years. However, when comparing climatic conditions at locations where *Hyalomma* specimens occurred vs. randomly generated pseudo-absence values within the same year, a significant effect of mean spring temperature became evident. It is believed that a critical threshold temperature is necessary to activate the molting of engorged nymphs; this has been cited as 15 ℃ for *H. marginatum* [25 cited Emelianova 2006]. Moreover, it was shown under laboratory conditions that the duration of molting from nymphal to adult ticks is temperature dependent, lasting between approximately 20 days at 28 ℃ and 70 days at 18 ℃ for *H. marginatum* [55].

Previously, the critical temperature threshold for molt was not reached beyond the Mediterranean range of the species; therefore, it was believed that this was a rare event in the second half of the twentieth century [14].

Moreover, the two *Hyalomma* species have different tolerance levels of relative humidity [26, 27]. Although humidity is regarded as equally important as temperature for defining the climate niche of both species [56], the seasonal drought index was not significantly associated with the *Hyalomma* occurrence locations in the present study. As mentioned before, due to the limitations of the citizen science approach, it was not possible to compare the *Hyalomma* numbers across years, but higher rainfall might explain the lower number of findings in 2021 compared to the preceding years.

The adults from both species are able to survive over harsh winter conditions, as for instance observed in non-Mediterranean countries like Ukraine, Romania or Bulgaria [57, 58]. So far, no evidence of an establishment of any of the two species in Germany is available. Specimens appeared from May in any given year, which is in line with introduction by migratory birds.

Besides temperature and relative humidity, other abiotic factors may also play a role for the survival, like the soil structure. *Hyalomma* species are so-called hunting ticks, hiding in the soil and waiting there for potential hosts passing by to attack them [59]. Both species mainly occur in steppe and semi-desert landscapes, and therefore the soil structure of these landscape types may be more important than thought so far. For example, the western Transcaucasia and mountain pastures are the areas from which *H. rufipes* has been reported, but not from valleys [4].

*Hyalomma marginatum* distribution includes southern Europe and northern Africa, while *H. rufipes* is an African species [28], with some reports outside Africa. In the present study, *H. marginatum* was found in all regions of Germany and in relatively higher percentage than *H. rufipes*, which might be explained by a better tolerance of local weather conditions by *H. marginatum* or an increased number of introductions from both Southern Europe by medium-distance migratory birds (moving within one or several European countries) and Africa by long-distance migratory birds (moving between Europe and Africa) at the same time. Moreover, differences in the reported number of ticks between federal states might be interpreted as an introduction following the west migration route, as shown in Chitimia-Dobler et al. [16] based on *R. aeschlimannii* analysis. In the Lazio Region in Central Italy, *H. marginatum* (27.7%), *H. rufipes* (51.8%), *Hyalomma* spp. (12.4%) and rarely *Amblyomma* spp. (3.6%), *I. ricinus* (0.7%) and *Ixodes* spp. (3.6%) were identified most frequently on 41 birds belonging to 17 species during the spring and autumn seasons [60]. This shows that many *H. rufipes* are carried by migratory birds, but weather conditions might be suboptimal for the development of *H. rufipes* nymphs into adults in many regions in Germany.

In the Northern Hemisphere, ticks transmit the largest number of zoonotic agents [61], wherefore the impact of climate change on ticks is of relevance within a One Health context. All *Hyalomma* tested negative for CCHV and *Babesia*/*Theileria* species. However, *R. aeschlimannii* circulates at a high percentage in the two *Hyalomma* species, as 44% tested positive. Nevertheless, *H. rufipes* tested significantly more frequently positive (21/43, 48.8%) for *R. aeschlimannii* compared to *H. marginatum* (30/132, 22.7%). A high prevalence of *R. aeschlimannii* (4/8, 50%) in *H. rufipes* was also observed in the samples from Germany in 2018 [16]. Furthermore, similar results were reported from Sweden [17]. Shuaib et al. [62] and Springer et al. [63] reported a high *Rickettsia* prevalence in *H. rufipes* in Sudan. As *R. aeschlimannii* belongs to the Spotted Fever Group of *Rickettsia* and has been associated with human infections, the results of the present study have an important relevance for public health. *Rickettsia aeschlimannii* was first isolated from *H. marginatum* ticks collected in Morocco in 1997 [64], while the first human *R. aeschlimannii* infection was documented in France in a traveler returning from Morocco in August 2000 [65]. Only one sequence was identify as *R. slovaca*. The explanation for this finding is that the *H. marginatum* was collected from a horse in Baden-Württemberg region, where the vector of *R. slovaca* is present, *Dermacentor marginatus* [66].

## Conclusions

While *Hyalomma* ticks were only sporadically found in Germany up to 2018, ticks of this genus have been reported every year from animals and humans in Germany since, with highly differing numbers between years. Locations of *Hyalomma* occurrence were associated with higher than average temperatures in the respective spring seasons. The continuous development of bird-imported nymphs into adult stages and their observed activity over 5 years warrant intensified and continuous surveillance.

### Supplementary Information

**Additional file 1. **

**Additional file 2. **

## Data Availability

Data supporting the conclusions of this article are included within the article and the sequences were submitted in GenBank.

## Abbreviations

CCHF: Crimean Congo hemorrhagic fever
PCR: Reverse transcription-polymerase chain reaction
ML: Maximum likelihood
