# Supplementary material for: Molting incidents of Hyalomma spp. carrying human pathogens in Germany under different weather conditions
Source: Parasit Vectors. 2024 Feb 19;17:70. doi: 10.1186/s13071-024-06175-y (PMC10877930; doi:10.1186/s13071-024-06175-y)
Supplement: Supplementary file 1 — Additional file 1. [file 13071_2024_6175_MOESM1_ESM.pdf]

Table S1: Output of generalized linear models with binomial error structure testing the relationship of climate variables with *Hyalomma* spp. occurrence vs. pseudo-absence in Germany during 2018, 2019 and 2020. For each year, ten sets of 1000 pseudo-absence values were randomly generated for the comparison. *P*-values < 0.05 are printed in bold.

	2018[a]				2019[b]				2020[c]			
	Estimate	Std. Error	z-value	*P*-value	Estimate	Std. Error	z-value	*P*-value	Estimate	Std. Error	z-value	*P*-value
**Replicate 1**												
Intercept	-3.79	6.26	-0.61	0.545	-7.11	2.99	-2.37	**0.018**	-7.25	4.63	-1.57	0.117
Elevation	0.00	0.00	-1.38	0.167	0.00	0.00	1.47	0.141	0.00	0.00	0.50	0.619
Mean spring temperature	0.62	0.43	1.44	0.149	1.20	0.33	3.60	**<0.001**	0.85	0.25	3.46	**0.001**
Spring drought index	0.24	0.14	1.70	0.090	-0.01	0.05	-0.18	0.856	0.05	0.15	0.36	0.721
Mean summer temperature	-0.36	0.45	-0.80	0.422	-0.35	0.25	-1.41	0.160	-0.18	0.31	-0.59	0.553
Summer drought index	-0.15	0.20	-0.78	0.438	0.01	0.08	0.13	0.899	-0.13	0.09	-1.52	0.129
**Replicate 2**												
Intercept	-5.62	6.45	-0.87	0.384	-5.13	3.02	-1.70	0.090	-11.14	4.75	-2.35	**0.019**
Elevation	0.00	0.00	-1.26	0.207	0.00	0.00	1.71	0.087	0.00	0.00	0.23	0.817
Mean spring temperature	0.53	0.44	1.21	0.225	1.48	0.35	4.20	**<0.001**	0.80	0.25	3.26	**0.001**
Spring drought index	0.23	0.14	1.58	0.114	-0.02	0.05	-0.44	0.664	0.05	0.15	0.30	0.762
Mean summer temperature	-0.23	0.47	-0.49	0.623	-0.58	0.26	-2.19	**0.029**	0.03	0.31	0.09	0.926
Summer drought index	-0.09	0.18	-0.50	0.616	-0.01	0.08	-0.12	0.908	-0.06	0.08	-0.79	0.432
**Replicate 3**												
Intercept	-4.25	6.21	-0.69	0.494	-5.06	3.13	-1.62	0.106	-9.29	4.79	-1.94	0.052
Elevation	0.00	0.00	-1.55	0.120	0.00	0.00	1.87	0.062	0.00	0.00	0.00	0.998
Mean spring temperature	0.61	0.43	1.44	0.149	1.49	0.34	4.39	**<0.001**	0.85	0.26	3.33	**0.001**
Spring drought index	0.26	0.14	1.82	0.068	-0.03	0.05	-0.56	0.579	0.11	0.15	0.69	0.488
Mean summer temperature	-0.34	0.44	-0.76	0.448	-0.58	0.26	-2.23	**0.026**	-0.10	0.33	-0.30	0.763
Summer drought index	-0.14	0.19	-0.74	0.459	-0.02	0.08	-0.22	0.829	-0.09	0.08	-1.05	0.292
**Replicate 4**												
Intercept	-5.68	6.25	-0.91	0.363	-6.73	3.16	-2.13	**0.033**	-9.02	4.55	-1.98	**0.048**
Elevation	0.00	0.00	-1.39	0.165	0.00	0.00	1.57	0.116	0.00	0.00	-0.01	0.990
Mean spring temperature	0.57	0.43	1.31	0.189	1.37	0.35	3.85	**<0.001**	0.88	0.25	3.51	**<0.001**
Spring drought index	0.24	0.14	1.69	0.090	-0.01	0.05	-0.25	0.803	0.02	0.16	0.16	0.873
Mean summer temperature	-0.24	0.45	-0.53	0.598	-0.44	0.27	-1.64	0.100	-0.12	0.31	-0.38	0.706
Summer drought index	-0.13	0.19	-0.68	0.495	-0.01	0.08	-0.19	0.849	-0.06	0.08	-0.73	0.464
**Replicate 5**												
Intercept	-5.90	6.33	-0.93	0.352	-6.49	3.02	-2.15	**0.032**	-7.27	4.83	-1.51	0.132
Elevation	0.00	0.00	-1.32	0.188	0.00	0.00	1.29	0.197	0.00	0.00	0.18	0.859
Mean spring temperature	0.54	0.44	1.21	0.226	1.39	0.36	3.86	**<0.001**	0.89	0.26	3.47	**0.001**
Spring drought index	0.21	0.14	1.51	0.131	0.01	0.06	0.13	0.896	0.01	0.16	0.09	0.929
Mean summer temperature	-0.20	0.45	-0.45	0.654	-0.47	0.27	-1.77	0.077	-0.19	0.33	-0.60	0.550
Summer drought index	-0.13	0.20	-0.67	0.504	-0.01	0.08	-0.11	0.915	-0.12	0.09	-1.38	0.168

**Replicate 6**												
Intercept	-4.17	6.40	-0.65	0.514	-6.27	3.10	-2.02	**0.043**	-6.92	4.76	-1.46	0.146
Elevation	0.00	0.00	-1.30	0.193	0.00	0.00	2.06	**0.040**	0.00	0.00	0.40	0.693
Mean spring temperature	0.63	0.44	1.45	0.147	1.53	0.34	4.47	**<0.001**	0.95	0.27	3.60	**<0.001**
Spring drought index	0.19	0.14	1.35	0.178	-0.03	0.05	-0.56	0.573	-0.03	0.15	-0.19	0.851
Mean summer temperature	-0.34	0.46	-0.74	0.461	-0.55	0.26	-2.13	**0.034**	-0.25	0.33	-0.74	0.457
Summer drought index	-0.13	0.19	-0.65	0.518	0.01	0.08	0.13	0.899	-0.10	0.08	-1.26	0.208
**Replicate 7**												
Intercept	-6.46	6.23	-1.04	0.300	-5.08	3.06	-1.66	0.097	-9.66	4.55	-2.12	**0.034**
Elevation	0.00	0.00	-1.14	0.256	0.00	0.00	1.93	0.054	0.00	0.00	0.32	0.751
Mean spring temperature	0.59	0.44	1.33	0.182	1.61	0.36	4.50	**<0.001**	0.77	0.25	3.10	**0.002**
Spring drought index	0.21	0.14	1.46	0.143	-0.04	0.05	-0.75	0.451	0.06	0.15	0.42	0.677
Mean summer temperature	-0.20	0.45	-0.44	0.658	-0.64	0.27	-2.41	**0.016**	-0.03	0.31	-0.09	0.926
Summer drought index	-0.15	0.20	-0.78	0.438	0.03	0.08	0.32	0.750	-0.09	0.08	-1.18	0.240
**Replicate 8**												
Intercept	-6.82	6.44	-1.06	0.290	-5.92	2.99	-1.98	**0.048**	-9.29	4.59	-2.02	**0.043**
Elevation	0.00	0.00	-1.64	0.101	0.00	0.00	1.36	0.174	0.00	0.00	0.01	0.992
Mean spring temperature	0.66	0.43	1.53	0.127	1.40	0.35	4.04	**<0.001**	0.81	0.25	3.28	**0.001**
Spring drought index	0.27	0.14	1.90	0.057	0.00	0.05	-0.04	0.971	0.08	0.15	0.55	0.580
Mean summer temperature	-0.25	0.46	-0.54	0.593	-0.51	0.26	-1.94	0.053	-0.07	0.31	-0.21	0.831
Summer drought index	-0.08	0.19	-0.40	0.692	0.02	0.08	0.22	0.826	-0.10	0.08	-1.25	0.212
**Replicate 9**												
Intercept	-5.33	6.51	-0.82	0.413	-5.10	2.97	-1.72	0.086	-6.78	4.62	-1.47	0.142
Elevation	0.00	0.00	-1.33	0.183	0.00	0.00	1.65	0.099	0.00	0.00	0.00	0.998
Mean spring temperature	0.53	0.45	1.18	0.238	1.39	0.34	4.09	**<0.001**	0.80	0.25	3.15	**0.002**
Spring drought index	0.25	0.14	1.74	0.082	0.00	0.05	0.05	0.960	0.09	0.16	0.60	0.548
Mean summer temperature	-0.24	0.48	-0.50	0.615	-0.54	0.25	-2.12	**0.034**	-0.19	0.32	-0.59	0.559
Summer drought index	-0.14	0.20	-0.69	0.488	-0.04	0.08	-0.52	0.602	-0.13	0.08	-1.57	0.117
**Replicate 10**												
Intercept	-4.77	6.37	-0.75	0.454	-6.84	3.05	-2.24	**0.025**	-5.69	4.77	-1.19	0.232
Elevation	0.00	0.00	-1.41	0.158	0.00	0.00	1.89	0.058	0.00	0.00	0.33	0.739
Mean spring temperature	0.55	0.43	1.29	0.196	1.56	0.35	4.49	**<0.001**	0.90	0.27	3.40	**0.001**
Spring drought index	0.23	0.14	1.64	0.101	-0.02	0.05	-0.37	0.711	-0.01	0.15	-0.09	0.927
Mean summer temperature	-0.28	0.45	-0.61	0.542	-0.54	0.26	-2.08	**0.038**	-0.28	0.33	-0.85	0.397
Summer drought index	-0.13	0.19	-0.68	0.497	0.00	0.08	-0.03	0.978	-0.13	0.08	-1.48	0.140

[a]: none of the models were significantly different from a null model containing only the intercept

[b]: all of the models were significantly different from a null model containing only the intercept

[c]: all of the models were significantly different from a null model containing only the intercept
