# Supplementary figures and images for: Molting incidents of Hyalomma spp. carrying human pathogens in Germany under different weather conditions
Source: Parasit Vectors. 2024 Feb 19;17:70. doi: 10.1186/s13071-024-06175-y (PMC10877930; doi:10.1186/s13071-024-06175-y)

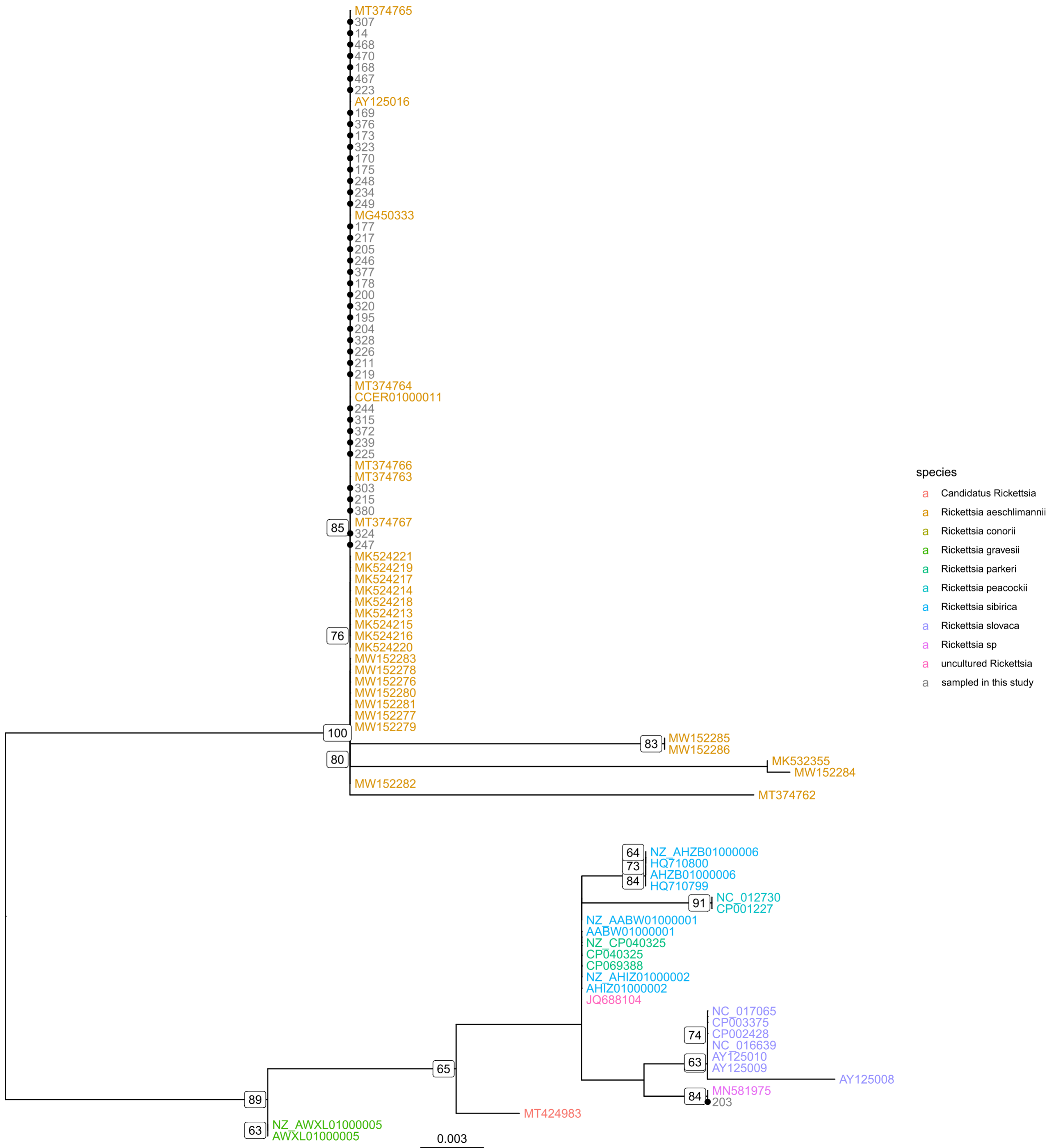

Supplement: Supplementary file 2 — Additional file 2. [file 13071_2024_6175_MOESM2_ESM.pdf]
